# Children of rural-to-urban migrant workers in China are at a higher risk of contracting severe hand, foot and mouth disease and EV71 infection: a hospital-based study

**DOI:** 10.1038/emi.2013.72

**Published:** 2013-10-23

**Authors:** Mei Zeng, Dongbo Pu, Xiaowei Mo, Chaomin Zhu, Sitang Gong, Yi Xu, Guangyu Lin, Beiyan Wu, Suli He, Xiaoyang Jiao, Xiangshi Wang, Xiaohong Wang, Qianqian Zhu, Ralf Altmeyer

**Affiliations:** 1 Department of Infectious Diseases, Children's Hospital of Fudan University, Shanghai 201102, China; 2 Institut Pasteur Shanghai, Chinese Academy of Sciences, Shanghai 200025, China; 3 Department of Infectious Diseases & Gastroenterology, Children's Hospital of Chongqing Medical University, Chongqing 400014, China; 4 Department of Infectious Diseases, Guangzhou Women and Children's Medical Center, Guangzhou 510120, China; 5 Pediatric Department of Shantou University Medical College, Shantou 515041, China

**Keywords:** EV71, hand, foot and mouth disease, migrant children, risk factor

## Abstract

The incidence and severity of hand, foot and mouth disease have increased in mainland China since 2008. Therapies and vaccines are currently at different stages of development. This study aimed to determine the social factors associated with the outbreaks and severity of the disease in Chinese children. A multicentre, prospective, case-controlled study was conducted in Shanghai, Chongqing, Guangzhou and Shantou to identify the sociodemographic and behavioural risk factors for hand, foot and mouth disease. Children hospitalized for hand, foot and mouth disease were randomly enrolled from April to November 2011. Stool samples were collected to test for the presence of enterovirus 71 (EV71). A total of 443 children between 1.6 and 68 months of age were enrolled; 304 were uncomplicated cases and 139 were severe cases with central nervous system involvement. The overall detection rate of EV71 was 54.2%, and the positivity rate of EV71 was significantly higher in the severe group than in the uncomplicated group (82.0% versus 40.9%, odds ratio (OR): 8.35, *P*=0.000). The children of migrant workers (OR: 3.014, *P*=0.000) and children attending kindergarten (OR: 2.133, *P*=0.002) were significantly associated with a severe outcome of the disease (OR: 1.765, *P*=0.026). Our findings indicate that kindergarten attendance and migrant worker parents are the major risk factors associated with severe hand, foot and mouth disease in children <5 years of age. Future public health intervention vaccination campaigns should consider the particular difficulties of achieving high compliance with multiple-dose vaccination regimens in the children of migrant workers.

## INTRODUCTION

The emergence and re-emergence of infectious diseases have been a major public health challenge in mainland China during the past decade.^1^ Hand, foot and mouth disease (HFMD) is a highly contagious infectious disease that primarily affects children under the age of 5 years. Severe disease with neurological complications and deaths mainly occur in children 1–4 years old. HFMD was occasionally reported in the early 1980s in China,^2^ but since the 2008 outbreak in Fuyang, Anhui Province, China, HFMD has become a notifiable disease.^3^ From January 2008 to 30 July 2012, more than 6.5 million children were diagnosed with HFMD and more than 2200 died in China. As of 30 August 2012, China had experienced a year-by-year increase of 66% in HFMD and 28% in mortality (http://www.moh.gov.cn). The data issued by the China Ministry of Health showed that HFMD ranked first among the notifiable infectious diseases in 2010 and 2012, and the deaths caused by HFMD ranked fifth.^4^ Similar to the situation in China, Vietnam and Singapore have witnessed consistently high activity of the disease. Recently, an outbreak of severe and fatal enterovirus 71 (EV71) infections occurred in Cambodia, leading to more than 60 deaths.^5^

The most common causative agent of severe HFMD is EV71, a virus belonging to the species human enterovirus A, genus *Enterovirus*, family Picornavirus. It is transmitted by the oral-faecal route and through contact with contaminated fluids and possibly through respiratory droplets. Following the 2008 outbreak, a series of guidelines for the prevention and management of HFMD were implemented in China, leading to an improved case-fatality ratio.^4^ Public education and public health measures do not have a significant effect on mitigating the outbreaks of HFMD in mainland China. Although no effective vaccine and therapeutic agents are currently available for the prevention and treatment of HFMD, a large number of candidates, which are at various stages from the preclinical to the late clinical stage, are under development.^6,7^ Large-scale nation-wide vaccination campaigns have been implemented successfully over the past decades in China for other viral diseases, e.g., hepatitis B and polio vaccination, leading to decreased mortality and morbidity.^8,9^

China's population is 1.37 billion people (November 2010), of whom one-half live in cities. In 2011, the Gross Domestic Product was estimated at 11.3 trillion US dollars, and the per capita Gross Domestic Product was 8382 US dollars. Economic growth has reduced the population living below the poverty line to 3% in 2011, from 10% in 2000. Improving health care has led to a reduction in infant mortality to 16/1000 in the year 2011 from 29/1000 in the year 2000. The employed population reached 780 million, including a rapidly growing population of 226 million migrant workers, who migrate predominantly from the rural central and western regions to the large coastal cities.^10^ Migrant workers frequently have lower education levels and less access to health care. Consequently, the weighted up-to-date and age-appropriate immunisation rates for childhood vaccines, such as the diphtheria, tetanus and pertussis combined vaccine, the oral poliomyelitis vaccine, the hepatitis B vaccine and the measles-containing vaccine, were significantly lower in the children of migrant workers than in those of the non-migrant population in the Beijing area.^11^

The purpose of this study is to assess the risk factors associated with EV71 infection and severe outcomes of HFMD in Chinese children, aimed at targeting the priority population in future nation-wide vaccination campaigns and providing insights for non-pharmaceutical intervention strategies for HFMD prevention. Future HFMD vaccine regimen and vaccination campaigns should address the particular nature of this high-risk paediatric population and the difficulty in achieving high coverage.

## MATERIALS AND METHODS

### Multicentre collaborative research network

We established a clinical research network in Shanghai (East China), Chongqing (Southwest China) and Guangzhou and Shantou (China). These three regions share common characteristics with respect to high population density and the subtropical maritime monsoon climate. Urbanisation is 89% in Shanghai, 82% in Guangzhou, 70% in Shantou and 55% in Chongqing. The participating hospitals included three provincial tertiary teaching paediatric hospitals (Shanghai Children's Hospital of Fudan University, Children's Hospital of Chongqing Medical University and Guangzhou Women and Children's Medical) and three paediatric departments of the general hospitals in Shantou (The First People Hospital, The Second People Hospital and Mingsheng Hospital). This study was reviewed and approved by the Ethics Committees of all hospitals.

A clinical co-investigator was assigned and trained at each centre before the study was performed. Approval for the study and for data sharing with the coordinating institution was granted by the institutional review boards at each participating institution.

### Case definitions and enrolment

HFMD is defined as oral vesicular exanthema/ulcers with vesicular lesions on the hands, and or feet, and or buttocks. A severe case was defined as HFMD accompanied with the occurrence of at least one of the following complications: aseptic meningitis, encephalitis, acute flaccid paralysis, pulmonary oedema or haemorrhage and cardiopulmonary collapse. All the diagnostic criteria of the clinical complications were consistent with the previously defined definition.^12^ Cerebrospinal fluid pleocytosis was defined as a white blood cell count of greater than 5×10^6^ cell/L in a patient older than 1 month of age. Migrant children were defined as having parents whose household registration was in a city different from the city where the parents live and work and where the child was enrolled; the parents of migrant children are almost always from rural areas.

### Inclusion criteria and data collection

The patients enrolled met the following inclusion criteria: (i) hospitalisation for HFMD; (ii) five years old or younger; (iii) residence in the local city at least six months before the disease onset; (iv) admission within four days of the onset of illness; and (v) informed consent to participate in this study from parents or guardians. We targeted enrolling 100 newly diagnosed admitted cases in each centre. HFMD cases were enrolled twice per week (Monday and Thursday) during the epidemic HFMD season from April to November 2011. Stool samples were collected from each study participant for virus detection. Each of the co-investigators completed a structured case report form on the detailed medical records, and all patient information was entreated into the Epidata program.

We collected and recorded the following information: the patient demographics (date of birth, date of presentation, sex and household registration), the clinical data (symptoms, clinical outcome, final clinical diagnosis, vaccination status, contact history, family background and social history), physical examination findings and laboratory test results (peripheral complete blood cell count and differential, peripheral glucose, cerebrospinal fluid test, electroencephalograph and brain imaging). All the data were submitted to the principal clinical investigator to determine the assignment of each patient into the uncomplicated or the complicated group, the latter representing the patients with neurological involvement.

### Identification of EV71

A commercially available EV71 diagnostic kit (Da An Gene Co., Ltd, Guangzhou, China) was used to detect EV71 in stool specimens. The detection method is based on a one-step real-time reverse transcription-polymerase chain reaction assay, and the sensitivity of the kit is 1×10^3^ plaque-forming units/mL. A specimen is considered positive for EV71 (EV71^+^) if the amplification curve crosses the threshold line within 35.1 cycles.

### Statistical analysis

The statistical analysis was performed using SPSS (version 11.5) software. The normally distributed data were compared using Student's *t*-test; the skewed distributed data were compared using the Mann–Whitney *U* test. The difference between the proportions was tested using the Chi-squared test with Yates' correction or Fisher's exact test. Univariate analysis was used to examine the association between the risk factors and complications of EV71 infection. A stepwise multiple logistic regression analysis was performed to adjust the confounders simultaneously and to calculate the multivariate-adjusted odds ratios (OR) for the risk factors. A *P* value <0.05 was considered to be significant.

## RESULTS

### Patients

We enrolled 443 children who met the inclusion criteria at the six participating hospitals from four regions as follows: 110 children were enrolled in Shanghai (63 uncomplicated cases and 47 severe cases), 120 children in Chongqing (61 uncomplicated cases and 59 severe cases), 98 children in Guangzhou (71 uncomplicated cases and 27 severe cases) and 115 children in Shantou (109 uncomplicated cases and 6 severe cases). Collectively, 304 (68.6%) were uncomplicated cases and 139 (31.4%) were severe cases complicated with meningitis or encephalitis, in which five patients progressed to pulmonary oedema and died.

### Demographic characteristics of patients

Among the 443 cases, boys and girls accounted for 65.5% and 34.5%, respectively (Table 1). No significant difference was found in the proportions of boys and girls between the uncomplicated group and the severe group (*P*>0.05). The five fatal cases were all boys.

The 443 children were aged between 1.6 and 68 months with the mean age of 25.9 months. The mean ages of the 304 uncomplicated cases and 139 severe cases were 25.1 months and 27.7 months, respectively (*P*>0.072). The highest number of cases was observed among the children 1–1.9 years old, accounting for 40.5% of the total cases, followed by 2-year-old children (25.7%), 3-year-old children (14.2%), 1-11 months-old infants (11.1%) and 4-5 years-old children (5.2%). There was no significant difference between the age groups and disease severity (*P*>0.05). Five deceased patients were 14–52 months old, and four were 1–1.9 years old.

### Vaccination record and underlying disease

Irregular or missed vaccinations were observed in the complicated group (13 cases) and the uncomplicated group (17 cases), according to parental reports or immunisation records. The Expanded Immunisation Program included vaccines for bacillus Calmette–Guérin (at birth), hepatitis B (at birth and one and six months), poliomyelitis (at two, three and four months), diphtheria, tetanus and pertussis combined vaccine (at three, four and five months), measles (at eight and 18 months) and Japanese encephalitis (at eight months) during infancy. Only four uncomplicated cases had an underlying disease with thalassanaemia, glucose-6-phosphate dehydrogenase deficiency, haemophilia and diabetes.

### Social background of patients

The exposure to disease, the model of child care, the household registration, residence and mother's education level were analysed. Collectively, prior exposure to HFMD was reported in 11.5% of the cases by parents. Although the proportion of children having prior exposure to HFMD cases was higher in the severe cases than in the uncomplicated cases (15.1% versus 9.9%), no significant difference was found (*P*=0.109). Local children and migrant children accounted for 70.9% and 29.1%, respectively, but severe disease occurred more frequently in migrant children than in local children (*P*=0.000). Children cared for at home and at kindergarten accounted for 76.5% and 23.5%, respectively, of the cases, but severe disease occurred more frequently in the children attending kindergarten (*P*=0.012). Children living in urban/suburban areas and in rural areas accounted for 70.5% and 29.5% of the severe cases, respectively. We did not identify a significant difference between residency and disease severity (*P*>0.05). Children whose mother had a primary to middle school education level constituted 40.9% of the cases, with severe disease more likely to occur than uncomplicated HFMD (49.6% versus 36.8%, *P*=0.011).

Univariate analysis identified three variables (kindergarten children, migrant children and education level of the mother) that were significantly associated with severe HFMD. These three parameters were included in a stepwise multivariate logistic regression analysis to adjust for possible confounds. This analysis identified that migrant children and kindergarten children represent an independent risk factor for severe HFMD (OR for migrant children: 3.014, *P*=0.000; OR for kindergarten children: 2.133, *P*=0.002).

### EV71 detection in patients

A total of 391 stool specimens were processed for EV71 detection (110 samples from Shanghai, 97 samples from Chongqing, 91 samples from Guangzhou and 93 samples from Shantou). Collectively, EV71 was detected in 212 (54.2%) of 391 cases, and the patients in all five fatal cases with pulmonary oedema were confirmed to be infected with EV71. Among the 52 patients from whom stool samples were not obtained, the ratio between the complicated and uncomplicated cases was 11–40, and the ratio between the migrant children and local children was 3–49.

As shown in Table 2, EV71 was detected more commonly in the severe cases than in the uncomplicated cases (39.2% (103/263) in the uncomplicated group and 85.2% (109/128) in the severe group (OR: 8.35, *P*=0.000). In Shanghai, 71.8% (79/110) of the patients were EV71^+^, of which 52.4% (33/63) were in the uncomplicated group and 97.9% (46/47) were in the severe group (*P*=0.000); in Chongqing, 82.5% (80/97) were EV71^+^, of which 77.1% (37/48) were in the uncomplicated group and 87.8% (43/49) were in the severe group (*P*=0.082); in Guangzhou, 29.7% (27/91) were EV71^+^, of which 17.2% (11/64) were in the uncomplicated group and 59.3% (16/27) were in the severe group (*P*=0.000); and in Shantou, 28.0% (26/93) were EV71^+^, of which 25% (22/88) were in the uncomplicated group and 80% (4/5) were in the severe group (*P*=0.008). EV71 infection was significantly more common among the severe cases versus the uncomplicated cases in Shanghai, Guangzhou and Shantou, whereas in Chongqing, no statistically significant difference of EV71 detection was found in the complicated versus the uncomplicated cases.

Three factors were identified as significantly correlated with EV71 infection, as follows: children with severe complications (OR: 8.58, *P*=0.000), migrant children (OR: 2.86, *P*=0.000), children having a prior exposure to an HFMD case (OR: 1.94, *P*=0.042) and children attending kindergarten (OR: 1.59, *P*=0.056). A stepwise univariate analysis confirmed migrant children and severe cases as independent risk factors for EV71 infection (OR for migrant children: 1.765, *P*=0.026; OR for severe cases: 7.825, *P*=0.000).

## DISCUSSION

HFMD is a highly contagious viral disease affecting young children, and non-pharmaceutical interventions have shown only a limited effect on the reduction of morbidity and mortality associated with severe disease.^13^ EV71 is the main virological factor that correlates with severe HFMD. Although greater than 80% of the severe cases are EV71^+^, not all EV71^+^ children develop severe disease.^14^ The clinical predictors for a severe outcome of HFMD are ≥three days of fever, peak temperature ≥38.5 °C, lethargy, hyperglycaemia, leukocytosis and limb weakness.^15,16^ Safe and effective pharmaceutical interventions are urgently needed to reduce the disease burden caused by HFMD. Understanding the target population for vaccination is crucial to developing appropriate vaccine candidates and vaccination campaigns that guarantee high compliance and protection. The present hospital-based study identified that in addition to kindergarten children, the children of migrant workers are at the highest risk of contracting EV71 infection and developing severe HFMD.

Our study confirmed previous findings that boys outnumbered girls, and young children aged 1–2.9 years accounted for the majority of complicated cases.^12,14^ Although boys and younger children are more susceptible to HFMD, gender and age were not related to the severity of disease. National data suggest that infants are at higher risk of severe disease than other age groups.^14^ This difference could be because of the regional variation of attack rates among age groups. In contrast to a study from Taiwan,^17^ which showed that living in rural areas was a risk factor of EV71 infection, we did not find an association between residence, severity of disease and EV71 infection.

Our results confirm that EV71 strongly correlates with severe disease and that EV71 strains are the major etiological agent of the 2011 HFMD epidemic in China that occurred after the 2008–2010 HFMD seasons.^18^ There are significant differences in the ratios of severe HFMD to all HFMD in four centres. Our data confirm earlier reports that EV71 is an independent high-risk factor of severe HFMD in China and that the detection rate of EV71 can predict the development of severe complicated enterovirus infection in Taiwan.^14,19^ Seroprevalence and immune protection against EV71 might be as high as 70% in the adult population, which positively affects protection in infants via maternal antibodies.^20^ As the maternal antibodies wane over the first months after birth, children become immune-naive to EV71 and susceptible to mild and severe infections, particularly between the ages of one and two years.^21^

Our study identified that children attending kindergarten are an independent risk factor for severe HFMD. We hypothesize that this finding is due to frequent and close contact with EV71-infected children. In this study, univariate analysis suggested that prior exposure was significantly associated with EV71 infection and that attending kindergarten is marginally associated with EV71 infection. We observed that the proportion of EV71-infected children with severe disease and reported that previous exposure is higher in children attending kindergarten than in home-cared for children. A previous study from Taiwan indicated kindergartens/day care centre attendance and prior exposure to HFMD cases as the risk factors for severe HFMD or EV71 infection.^17,21^ A series of infection control measures, such as morning check, routine disinfection of daily utensils, tools and environment surfaces, case isolation and class closure upon the appearance of cluster cases, have been strengthened in mainland China since the 2008 outbreak. Despite these measures, attending kindergarten is more likely to be associated with EV71 infection in mainland China. We hypothesize that symptomatic and asymptomatic EV71 infection play important roles in the transmission of HFMD among children in the kindergarten setting. On the basis of serologically confirmed EV71 infection, the proportions of asymptomatic EV71 infection were 71% and 86.7% after the HFMD outbreaks in Taiwan (1998) and in Shanghai (2010), respectively.^17,21^ Virus shedding in patients' stool can persist for 8 weeks after disease onset.^22^ Convalescent children with active viral shedding may have frequent exposure to other children in the kindergarten/day care centre setting.

This hospital-based study shows that having migrant worker parents is an independent risk factor for EV71 infection and severe outcome of HFMD in children <5 years old. This finding is important because the rural-to-urban migrant population in China has increased dramatically following the rapid economic development and urbanisation in China since the 1990s. The 2010 census revealed that the migrant worker population is 230 million.^10^ Population migration plays a critical role in the spread of infectious diseases and affects the emergence, re-emergence and changes in the prevalence of infectious diseases.^23,24^ The migrant population often faces greater difficulties in accessing appropriate health care for diagnosis and treatment because of a lack of knowledge about disease, which is a result of low education and poor economic conditions. We found that the low education levels of mothers correlated with the severity of HFMD. Knowledge about the disease is critical to providing early supportive care because a one-day delay in HFMD diagnosis was shown to result in an increase in the odds of progression to severe disease by 40% and in an increase in the odds of a fatal outcome by 54%.^14^ The findings of this study are based on hospitalized patients and do not include data from non-hospitalized cases, which could potentially introduce a bias. We hypothesize that massive rural-to-urban migration and dense population in cities in mainland China remain an important risk for the transmission of infectious diseases, particularly paediatric infections such as HFMD for which no vaccine or therapy is available. Further population-based studies are necessary to address the social factors associated with severe HFMD and EV71 epidemics.

### Conclusion

An understanding of the social factors related to severe HFMD is necessary to formulate practical strategies for infectious disease prevention and control. Better education of migrant workers on HFMD could further reduce the morbidity and mortality resulting from severe disease. Therapies and vaccines for EV71 are urgently needed, and migrant children should be considered the priority target population for vaccination. As is the case with the related poliovirus, a multiple-dose vaccination regimen over several weeks may be necessary. Such a vaccination regimen would face a significant challenge in light of the previously demonstrated low compliance and coverage of vaccination in the migrant worker population in China.

## Figures and Tables

**Table 1:** Sociodemographic characteristics and EV71 detection in children with HFMD

Characteristics	Enrolled cases (*n*=443)	Uncomplicated cases (*n*=304)	Severe cases (*n*=139)	OR	*P*
**Age (months)**					
Median	25.9	25.1	27.7		
<12	49 (11.1%)	38 (12.5%)	11 (7.9%)	0.60	0.153
12–23	180 (40.5%)	124 (40.8%)	56 (40.3%)	0.98	0.921
23–35	114 (25.7%)	81 (26.6%)	33 (23.7%)	0.86	0.517
36–47	63 (14.2%)	38 (12.5%)	25 (18.0%)	1.54	0.125
48–60	37 (8.4%)	23 (7.6%)	14 (10.1%)	1.37	0.376
**Gender**					
Boys	290 (65.5%)	201 (66.1%)	89 (64.0%)	0.91	0.668
Girls	153 (34.5%)	103 (33.9%)	50 (36.0%)	1.10	0.668
**Exposure history**					
Contact with cases	51 (11.5%)	30 (9.9%)	21 (15.1%)	1.63	0.109
**Household registration**					
Local	314 (70.9%)	233 (76.7%)	81 (58.3%)	0.43	0
Migrant	129 (29.1%)	71 (23.3%)	58 (41.7%)	2.35	0
**Child care**					
Home care	339 (76.5%)	243 (79.9%)	96 (69.1%)	0.56	0.012
Day care centre/kindergartens	104 (23.5%)	61 (20.1%)	43 (30.9%)	1.78	0.012
**Residence**					
Urban	169 (38.1%)	119 (39.1%)	50 (36.0%)	0.87	0.523
Suburb	166 (37.5%)	118 (38.8%)	48 (34.5%)	0.83	0.387
Rural	108 (24.4%)	67 (22.0%)	41 (29.5%)	1.48	0.09
**Mother's education**					
Elementary–middle school	181 (40.9%)	112 (36.8%)	69 (49.6%)	1.69	0.011
High or technical school	132 (29.8%)	98 (32.2%)	34 (24.5%)	0.68	0.097
College or above	130 (29.3%)	94 (30.9%)	36 (25.9%)	0.78	0.281
**EV71 infection**					
Shanghai	79/110 (71.8%)	33/63 (52.4%)	46/47 (97.9%)	41.8	0
Chongqing	80/97 (82.5%)	37/48 (77.1%)	43/49 (87.8%)	2.13	0.167
Guangzhou	27/91 (29.7%)	11/64 (17.2%)	16/27 (59.3%)	7.01	0
Shantou	26/93 (28.0%)	22/88 (25%)	4/5 (80%)	12	0.008
Four regions	212/391 (54.2%)	103/263 (39.1%)	109/128 (85.1%)	8.35	0

Abbreviations: EV71, enterovirus 71; HFMD, hand, foot and mouth disease; OR, odds ratio.

**Table 2:** Factors associated with EV71 infection in children with HFMD

Characteristics	EV71-infected cases (*n*=212)	Non-EV71-infected cases (*n*=179)	OR	*P*
**Severity of disease**				
Complicated	109 (51.4%)	19 (10.6%)	8.58	0
Uncomplicated	103 (48.6%)	160 (89.4%)	0.12	0
**Age (months)**				
Median	27.4	24.3		
<12	19 (9.0%)	22 (12.3%)	0.702	0.285
12–23	84 (39.6%)	76 (42.5%)	0.89	0.57
23–35	54 (25.5%)	48 (26.8%)	0.93	0.763
36–47	34 (16.0%)	23 (12.8%)	1.30	0.373
48–60	21 (9.9%)	10 (5.6%)	1.86	0.115
**Gender**				
Boys	133 (62.7%)	119 (66.5%)	0.85	0.441
Girls	79 (37.3%)	60 (33.5%)	1.18	0.441
**Exposure history**				
Contact with cases	32 (15.1%)	15 (8.4%)	1.94	0.042
**Household registration**				
Local	125 (59.0%)	138 (77.1%)	0.43	0.000
Migrant	87 (41.0%)	35 (22.9%)	2.86	0
**Child care**				
Home care	153 (72.2%)	144 (80.4%)	0.63	0.056
Day care centre/kindergartens	59 (27.8%)	35 (19.6%)	1.59	0.056
**Residence**				
Urban	74 (34.9%)	68 (38.0%)	0.88	0.528
Suburb	86 (40.6%)	60 (33.5%)	1.35	0.151
Rural	52 (24.5%)	51 (28.5%)	0.82	0.375
**Mother's education**				
Elementary–middle school	90 (42.5%)	72 (40.2%)	1.10	0.656
High or technical school	61 (28.8%)	56 (31.3%)	0.89	0.589
College or above	61 (28.8%)	51 (28.5%)	1.01	0.951

Abbreviations: EV71, enterovirus 71; HFMD, hand, foot and mouth disease; OR, odds ratio.
